# Detection of a Cis eQTL Controlling *BMCO1* Gene Expression Leads to the Identification of a QTG for Chicken Breast Meat Color

**DOI:** 10.1371/journal.pone.0014825

**Published:** 2011-07-05

**Authors:** Elisabeth Le Bihan-Duval, Javad Nadaf, Cécile Berri, Frédérique Pitel, Benoît Graulet, Estelle Godet, Sophie Y. Leroux, Olivier Demeure, Sandrine Lagarrigue, Cécile Duby, Larry A. Cogburn, Catherine M. Beaumont, Michel J. Duclos

**Affiliations:** 1 INRA, UR83, Recherches Avicoles, Nouzilly, France; 2 INRA, ENVT, UMR444 Génétique Cellulaire, Castanet-Tolosan, France; 3 INRA, UR1213 Herbivores, Centre de Clermont-Ferrand/Theix, St-Genès-Champanelle, France; 4 INRA, UMR 598 Génétique Animale, Rennes, France; 5 Agrocampus Ouest, UMR 598 Génétique Animale, Rennes, France; 6 Department of Animal and Food Sciences, University of Delaware, Newark, Delaware, United States of America; Leiden University Medical Center, Netherlands

## Abstract

Classical quantitative trait loci (QTL) analysis and gene expression QTL (eQTL) were combined to identify the causal gene (or QTG) underlying a highly significant QTL controlling the variation of breast meat color in a F2 cross between divergent high-growth (HG) and low-growth (LG) chicken lines. Within this meat quality QTL, *BCMO1* (Accession number GenBank: AJ271386), encoding the β-carotene 15, 15′-monooxygenase, a key enzyme in the conversion of β-carotene into colorless retinal, was a good functional candidate. Analysis of the abundance of *BCMO1* mRNA in breast muscle of the HG x LG F2 population allowed for the identification of a strong cis eQTL. Moreover, *r*eevaluation of the color QTL taking *BCMO1* mRNA levels as a covariate indicated that *BCMO1* mRNA levels entirely explained the variations in meat color. Two fully-linked single nucleotide polymorphisms (SNP) located within the proximal promoter of *BCMO1* gene were identified. Haplotype substitution resulted in a marked difference in *BCMO1* promoter activity in vitro. The association study in the F2 population revealed a three-fold difference in *BCMO1* expression leading to a difference of 1 standard deviation in yellow color between the homozygous birds at this haplotype. This difference in meat yellow color was fully consistent with the difference in carotenoid content (i.e. lutein and zeaxanthin) evidenced between the two alternative haplotypes. A significant association between the haplotype, the level of *BCMO1* expression and the yellow color of the meat was also recovered in an unrelated commercial broiler population. The mutation could be of economic importance for poultry production by making possible a gene-assisted selection for color, a determining aspect of meat quality. Moreover, this natural genetic diversity constitutes a new model for the study of β-carotene metabolism which may act upon diverse biological processes as precursor of the vitamin A.

## Introduction

For more than half of a century, commercial poultry breeding programs have focused mainly on improvements of two major production traits, growth rate and feed efficiency, in meat-type (broiler) chickens. Furthermore, different experimental lines of chickens have been created to increase our understanding of genetic control over other important production traits, including meat quality. Our unique model is a population of meat-type chickens that was divergently selected for high (HG) or low growth (LG) rate, based on a difference in body weight (BW) at both 8 and 36 weeks of age [1]. A genetic analysis of the highly heritable growth curve from this experimental selection has been described in detail [2], [3]. The HG and LG broiler lines have been extensively studied to understand the physiological and genetic basis of marked differences in growth rate and skeletal muscle development [4], [5]. An increase in fiber diameter and at a less extent in the total number of muscle fibers accounts for the greater breast and leg muscle weights of the HG birds [6]. Recently, we found that the HG chickens exhibit a paler meat characterized by higher lightness (BCo-L), lower redness (BCo-R) and yellowness (BCo-Y) than that of LG birds. Several QTL for meat quality were detected in the F2 resource population created from the HG x LG intercross, among these was a strong QTL on *GGA*11 that affects color of breast meat (i.e., yellowness and redness) [7]. Here, we describe the identification through a cis eQTL analysis of the causal gene and regulatory mutation underlying this meat quality QTL.

## Results

### Refinement of the Meat Color QTL Region

**Figure 1:**
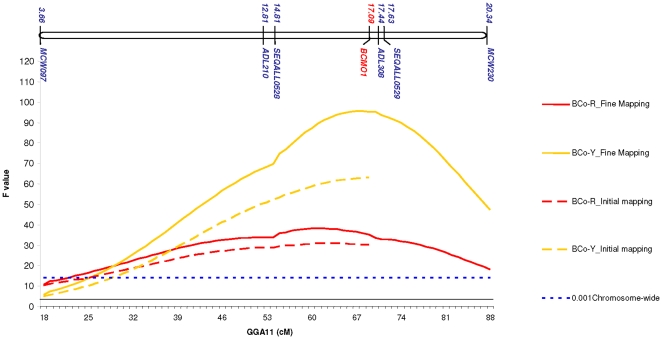
Relative position of the yellowness (BCo-Y) and redness (BCo-R) QTL and of the *BCMO*1 gene. F profiles show a better localization of the BCo-Y and BCo-R trait QTL by genetic map densification (solid lines) with six markers compared to initial mapping (dashed lines) with three markers (MCW097, ADL210 and ADL308). *BCMO1* gene is located on *GGA*11 between 17.085 and 17.105 Mbp, the approximate location of the observed BCo-Y QTL.

At the step of initial mapping, the position of the meat color QTL was imprecise, due to the lack of microsatellite markers on the distal end of *GGA*11. To refine the QTL location, three additional markers were developed and the QTL re-analyzed. As shown in Figure 1, the QTL for the breast muscle yellowness (BCo-Y) trait was detected at about the same position as with the initial mapping (at 68 cM *vs*. 69 cM), although the significance level was higher (F statistic of 95 *vs.* 63). In addition, the confidence interval of the QTL was reduced from 35 (13.3–21.9 Mb) to 17 cM (14.4–18.4 Mb). The origin of the high allele for BCo-Y was traced back to the LG line, which was consistent with the more intense yellow color of breast meat in this genotype. The QTL on *GGA*11 explained about 14% of the phenotypic variance in the BCo-Y trait in the HG x LG F2 population. Half-sib analysis showed that all F1 sires were heterozygous at this QTL, which suggested fixation of alternative alleles in the founder lines originally created from a mixing of several commercial breeds. Similar improvements in refinement of the QTL were obtained for the breast muscle redness (BCo-R), although the level of significance for this trait remained lower. The QTL region for the breast muscle redness trait (BCo-R) overlapped that for yellowness trait (BCo-Y). These observations suggested a strong impact of the *GGA*11 QTL on the variation of meat color observed between the HG and LG lines.

### Identification through eQTL Analysis of the Causal Gene

**Figure 2:**
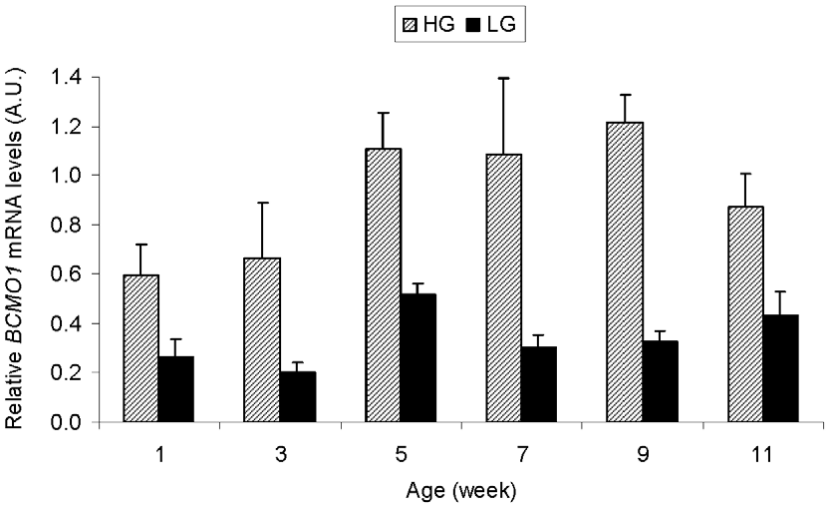
Relative *BCMO1* mRNA levels in the HG and LG lines according to age. The relative *BCMO1* mRNA levels (2^(dCT BCMO1-dCT 18S)^) have been measured in the *Pectoralis major* muscle of HG and LG chickens slaughtered at 1, 3, 5, 7, 9, and 11 weeks of age (n = 6 by line/age). Age and line effects were tested by two-way analysis of variance. A highly significant effect of the line (P<0.0001) was evidenced, without any interaction with age.

**Figure 3:**
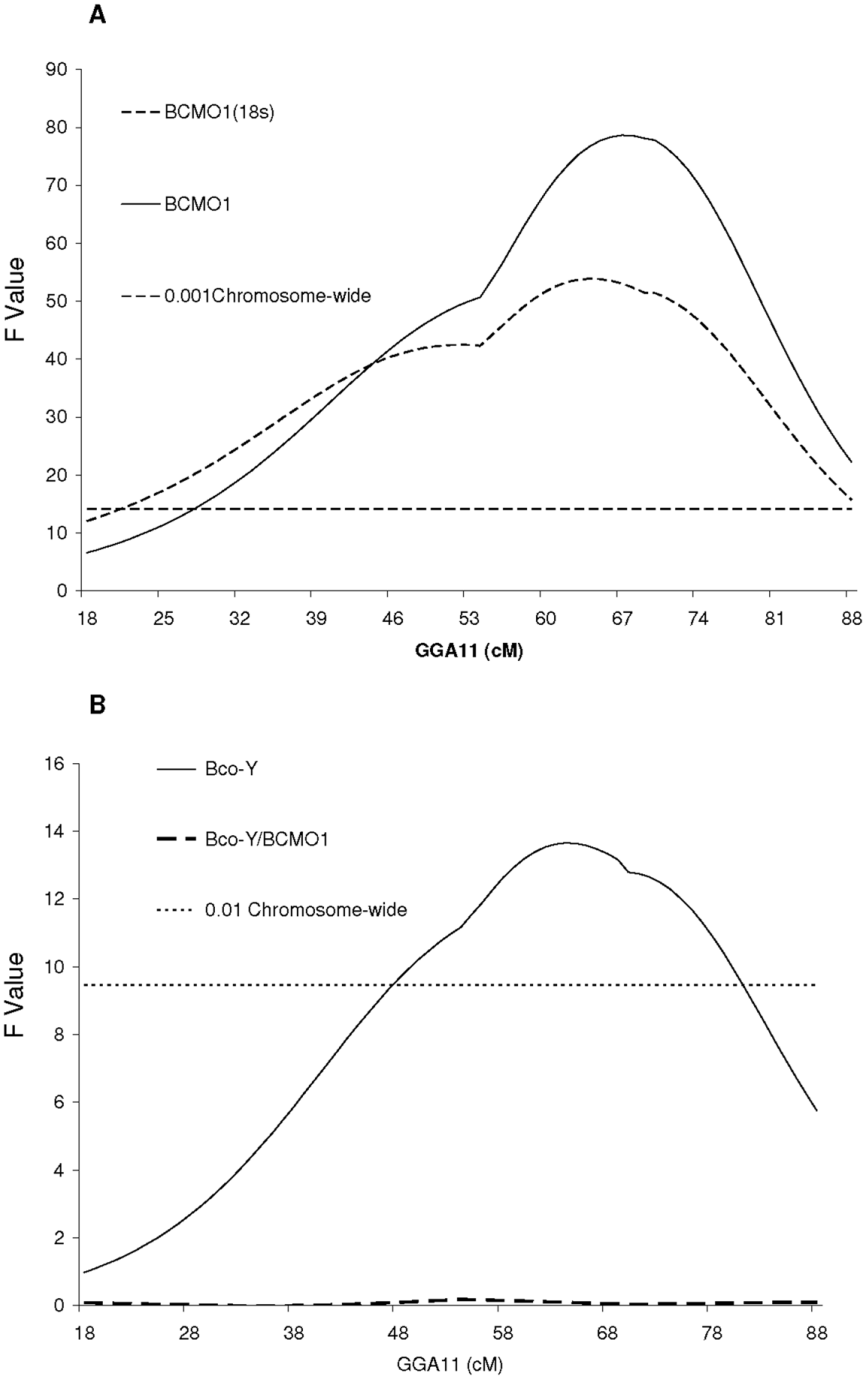
Identification of an eQTL controlling *BCMO1* mRNA levels. (**a**) A strong cis eQTL for absolute (dCT BCMO1, solid line) or relative (dCT BCMO1-dCT 18S, dashed line) *BCMO1* mRNA levels is detected on GGA11 (**b**). The QTL for BCo-Y (solid line) completely disappeared (dashed line) when *BCMO1* mRNA level (dCT BCMO1) was considered as covariate in the model.

Refinement of this meat quality QTL region reduced the number of positional candidate genes from about 140 in the initial mapping to 30 genes. The most obvious candidate gene was *BCMO1*, being located on *GGA*11 between 17.085 and 17.105 Mbp, the approximate location of the observed BCo-Y QTL (Figure 1). Furthermore, *BCMO1* is a good functional candidate because it encodes β-carotene 15, 15′-monooxygenase, an enzyme responsible for the conversion of β-carotene (a yellow pigment) into two colorless retinal (pro-vitamin A) molecules [8]. We first compared *BCMO1* mRNA levels in the breast muscle of HG and LG birds across six ages (1-11 wk). As reported in Figure 2, the level of *BCMO1* mRNA was consistently higher in the *Pectoralis major* muscle of HG chickens compared to LG chickens, regardless of age. This large difference in abundance of *BCMO1* transcripts between LG and HG birds was evident with or without normalization to 18S ribosomal RNA levels. We then examined the relationship between variations of *BCMO1* mRNA levels and the yellowness of breast meat (BCo-Y) in the segregating HG x LG F2 population. Breast muscle *BCMO1* mRNA levels were quantified in one of the five F1 half-sib families (n = 134). A significant negative correlation (r =  −0.47) between *BCMO1* mRNA levels and the BCo-Y measurements was observed. Subsequently, a QTL analysis was performed on *GGA*11 using *BCMO1* mRNA levels as a quantitative trait. As shown in Figure 3a, a strong *cis* expression QTL (eQTL) was detected that was even more significant than the original BCo-Y QTL (Fig. 3b; F value of 79 *vs.* 14). The high allele for the eQTL (*BCMO1* mRNA) was traced back to the HG line, indicating that higher expression of the *BCMO1* gene is linked to lower meat yellowness in HG birds. The QTL analysis for the BCo-Y trait was also completed using breast muscle *BCMO1* mRNA levels as a covariate. As illustrated by Figure 3b, the QTL for BCo-Y previously detected on *GGA*11 disappeared after this correction. Collectively, these observations clearly show that the variation in the BCo-Y trait is linked to the variation of *BCMO1* mRNA levels in breast muscle.

### Gene Sequencing and Genotyping of Putative Causative SNPs in the F2 Population

**Figure 4:**
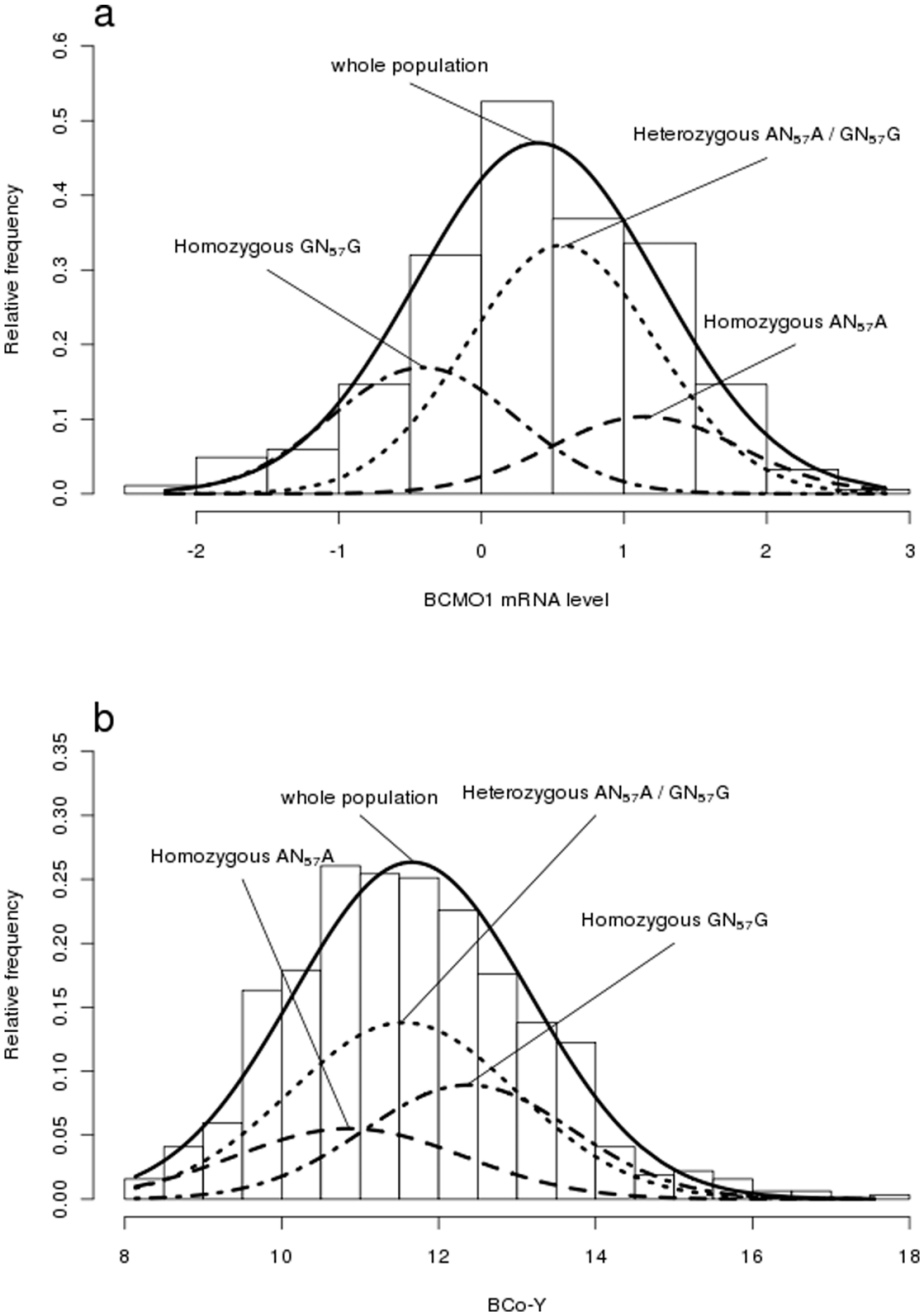
Association analyses between *BCMO1* haplotype and *BCMO1* expression or breast meat color. An examination of the F2 population (N = 373) demonstrated a significant effect of the haplotype on *BCMO1* mRNA levels (dCT *BCMO1*) (**a**) and BCo-Y parameter (**b**). The heterozygous birds (AN_57_A/GN_57_G) showed intermediate mRNA levels and BCo-Y values.

Next, we sequenced the promoter region and 11 exons of the *BCMO1* gene to identify mutations underlying variations in *BCMO1* transcript levels. The causal mutation was expected to be heterozygous in the five F1 sires, which were all heterozygous for BCo-Y QTL. Amplified fragments of the *BCMO1* gene were sequenced in the five F1 sires from the HG x LG intercross, where a total of 23 SNP and 4 insertion/deletion polymorphisms were identified in the *BCMO1* gene and its promoter. Six of the SNP observed in our population are publically available (http://genome.ucsc.edu/cgi-bin/hgGateway: snp.37.663.6748.S.3, snp.37.663.6897.S.3, snp.37.663.6906.S.3, snp.37.663.10743.S.2, snp.37.663.10815.S.2, snp.37.665.1391.S.3). Twenty-one new polymorphisms were observed (accession numbers ss325995734 to ss325995754). Six of the 23 SNP were heterozygous for all F1 sires: one semi-conservative SNP (Glu>Lys, AA residue 524) was within exon 11, without any supposed effect on *BCMO1* mRNA level, two SNP were within intron 8, one within intron 9 and, interestingly, two SNPs within the promoter region. Since the QTL controlled the *BCMO1* gene expression and since the promoter is known as a region regulating expression, the last two SNPs positioned at −678 (SNP1) and −621 (SNP2) upstream of ATG (Figure S1) were considered as the most likely candidate mutations. A genotyping assay, based on high-resolution melting (HRM) curve analysis, was developed to test the association between the SNP1 and SNP2 mutations and variations in *BCMO1* mRNA levels and meat color traits (BCo-Y and BCo-R) in the HG x LG F2 population. The two SNPs in the *BCMO1* promoter were fully linked defining two AN_57_A and GN_57_G haplotypes. No recombinant AN_57_G or GN_57_A haplotype was observed in any of the F0, F1 or F2 birds. Genotyping of 35 F0 birds showed a difference in allele frequencies between the two lines. The GN_57_G haplotype was fixed in F0 LG birds (N = 20) while the AN_57_A haplotype was predominant in F0 HG birds (9 of 13 birds tested had the AN_57_A type). As shown in Figure 4a, an examination of the F2 population (N = 373) demonstrated a significant effect of the haplotype on *BCMO1* mRNA levels. Least square means (LSM) for mRNA levels were estimated at 1.17 (N = 63) and -0.48 (N = 101) in homozygous AN_57_A and GN_57_G birds, which corresponded to a 3 fold higher abundance of *BCMO1* transcripts in the AN_57_A birds than in the GN_57_G birds. The two haplotypes acted additively, the heterozygous birds (AN_57_A/GN_57_G) showing intermediate mRNA levels (LSM of 0.56, N = 203). As shown by Figure 4b, a similar and consistent feature was observed for variation in yellow color of breast muscle trait (BCo-Y). A difference of 1.5 BCo-Y units (∼1 S.D.) was found between the extreme homozygous birds, while the heterozygous birds were intermediate [LSM of 10.9 (N = 128), 11.6 (N = 318) and 12.4 (N = 188) for the homozygous AN_57_A, the heterozygous AN_57_A/GN_57_G, and the homozygous GN_57_G, respectively]. A lower but significant effect was found for the red color of the meat with a difference of 0.6 unit of BCo-R (∼0.75 S.D.) between extreme homozygous genotypes, the heterozygous genotype being intermediate [LSM of 0.7 (N = 129), 1.1 (N = 320) and 1.3 (N = 190) for the homozygous AN_57_A, the heterozygous AN_57_A/GN_57_G, and the homozygous GN_57_G, respectively].

### Impact of the haplotype on breast muscle carotenoid contents and relationship with breast meat color

**Figure 5:**
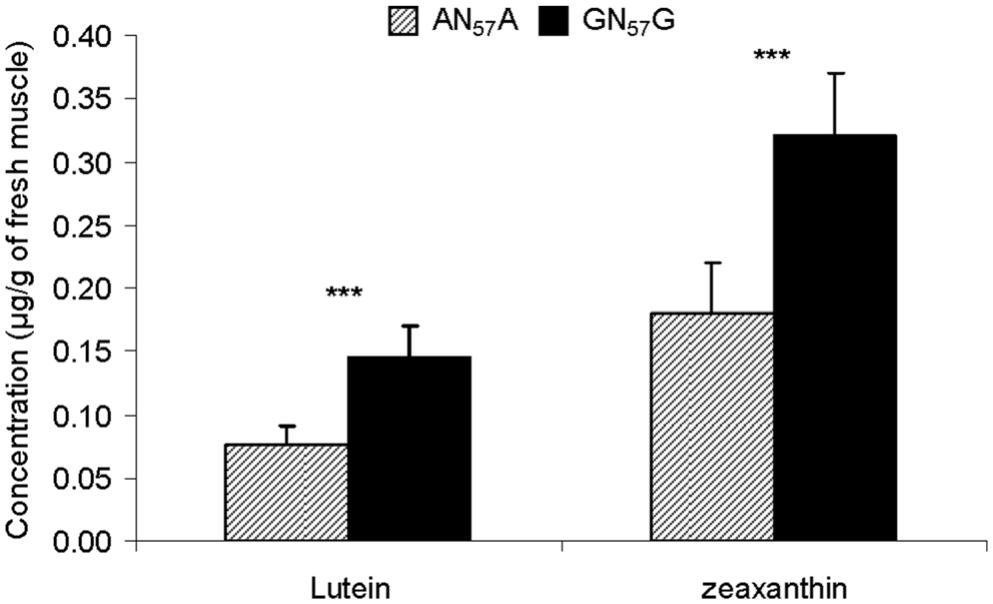
Effect of the *BCMO1* haplotype on carotenoid pigment content of *Pectoralis major* muscle. ***, P<0.001 Carotenoid concentration and composition were determined through extraction, purification and chromatographic analysis using UPLC in *Pectoralis major* muscle samples from populations of chicken with the two linked SNP (n = 11 for GN_57_G and n = 9 for AN_57_A). Lutein and zeaxanthin were the sole carotenoids detected in the samples. Their concentrations were 1.9 and 1.8-fold higher for lutein and for zeaxanthin, respectively, in GN_57_G than in AN_57_A chicken (P<0.001).

The impact of the haplotype on breast muscle carotenoid content was evaluated. Only lutein and zeaxanthin pigments were detected in breast muscle, while β-carotene was undetectable. Carotenoid content was markedly higher in GN_57_G (n = 11) than in AN_57_A (n = 9) with a ratio of 1.9 and 1.8 for lutein and zeaxanthin, respectively (Figure 5). Across all samples (n = 20) a significant positive relationship was observed between the total content in carotenoids (lutein + zeaxanthin) and the yellow color of breast meat (Pearson correlation, R  =  +0.6).

### Screening and effect of the mutation in a commercial chicken population

**Figure 6:**
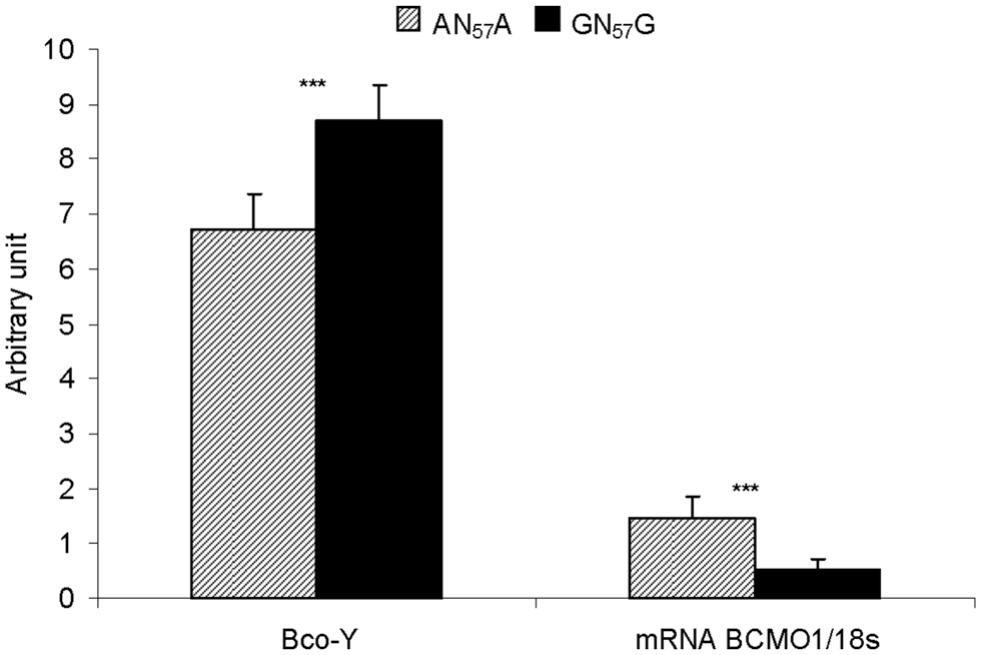
Effect of the *BCMO1* haplotype in an unrelated commercial population. Breast meat yellowness (BCo-Y) and relative *BCMO1* mRNA level (2^(dCT BCMO1-dCT 18S)^) were measured in the *Pectoralis major* muscle of birds homozygous at the *BCMO*1 locus (n = 11 for AN_57_G and n = 12 for GN_57_G). BCo-Y value was significantly lower (***, P<0.001) while *BCMO1* mRNA level was higher (***, P<0.001) in AN_57_G than in GN_57_G.

Five out of the ten sires from a commercial population of slow-growing broilers used for “Label Rouge” production were found to be heterozygous for the *BCMO1* haplotype. As for the experimental population, the association study performed in the commercial population showed a significant effect of the haplotype on the color of the breast meat, with differences of 0.5 unit of b* (p = 0.007) and 0.4 unit of a* (p = 0.006) between the homozygous birds (LSM of 8.5 and 8.0 for the b* color of the homozygous GN_57_G and AN_57_A respectively; LSM of −1.3 and −1.7 for the a* color of the homozygous GN_57_G and AN_57_A respectively). The effect of the haplotype on *BCMO1* expression was evaluated in a sample of 11 AN_57_A and 12 GN_57_G homozygous birds. *BCMO1* mRNA levels were three-fold higher while BCo-Y values were significantly lower in AN_57_A compared to GN_57_G (Figure 6).

### The Two Fully Linked SNPs have a Functional Effect on the Promoter Activity

**Figure 7:**
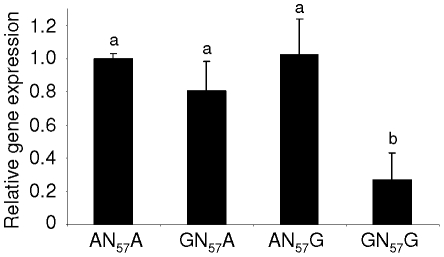
Effect of AN_57_A, GN_57_G, and “recombined”AN_57_G and GN_57_A haplotypes on pGL2-BCMO1-Luc luciferase activity. LMH cells were transiently transfected with 200 ng of a luciferase reporter containing sequences from −734 to +97 bp of the chicken *BCMO1* promoter and 20 ng of a reference plasmid containing the β-galactosidase gene. For each condition, n = 3. Luciferase activities normalized to β-galactosidase activity are relative to the AN_57_A condition. AN_57_A, GN_57_A and AN_57_G had similar effects on luciferase expression while GN_57_G was associated with a significantly lower expression (P<0.05).

The functional effect of the wild type (AN_57_A and GN_57_G) and recombinant (GN_57_A and AN_57_G) haplotypes on the promoter activity was further investigated using a gene reporter strategy. As the AN_57_A, GN_57_A and AN_57_G haplotypes were obtained by mutagenesis of the GN_57_G haplotype, the gene expression variation can directly be associated to the two targeted SNPs. Chicken hepatoma cells (LMH) were transfected with a luciferase reporter construction containing a −734 to +97 fragment of the chicken *BCMO1* promoter, corresponding to a unique sequence except for the two candidate SNPs. The AN_57_A construction was associated with a 3.7 fold higher luciferase activity than the GN_57_G construction (Figure 7). Consistent results were obtained using human hepatoma cell lines (data not shown). For both GN_57_A and AN_57_G constructions, the luciferase activity was similar to the AN_57_A haplotype but significantly higher than the one observed with the GN_57_G haplotype (Figure 7). These results were confirmed in three independent experiments and demonstrated that both SNP are required to affect the activity of *BCMO1* promoter and consequently the level of *BCMO1* transcripts.

## Discussion

While several hundreds QTL have been reported in livestock species including chicken (http://www.animalgenome.org/QTLdb), the identification of the underlying genes and mutations is still exceptional [9], [10]. This study is a good example of the strength of combining positional and functional data since the eQTL approach validated the choice of a candidate gene and rapidly gave strong indications about the underlying mutation(s). Our study supports the conclusion that *BCMO1* is the gene underlying the chicken meat color QTL on GGA11, through a cis eQTL controlling its expression. Two fully linked SNPs in the *BCMO1* promoter defined two haplotypes (AN_57_A and GN_57_G) with respectively high and low promoter activity as proven in vivo and in vitro. Genomatix was used to check if the mutations could alter a transcription factor binding site. If no results were obtained for the first mutation, the second mutation alters a Vitamin D receptor (VDR), a DR5 binding site detected only with the A allele. However, we clearly demonstrated that both mutations are needed to modify the gene expression. This could be due either to a transcription factor binding site undetected in the first mutation region or most likely to an interaction between the two mutations, one hypothesis being that the first mutation alters the genome 3D structure and affects the fixation of transcription factors in this region. These variations are likely to impact on the biological activity of the *BCMO1* enzyme as they lead to respectively low or high carotenoid content in breast meat. Of the major food quality attributes, color is the first critical one since it is the main visual factor involved in the consumer's choice [11]. The identified *BCMO1* polymorphism mainly affected the yellow color of breast meat, by altering the content of the yellow pigments lutein and zeaxanthin. These two carotenoids, although not considered as substrates of the *BCMO1* enzyme [12], could be converted into β-carotene before symmetric cleavage by *BCMO1,* as already described for related xanthophylls (canthaxanthin and astaxanthin) in fish [13] and in rat [14]. The effect of *BCMO1* polymorphism on red color was less marked, likely because redness may be influenced by other mechanisms such as muscle blood content at death. This last parameter depends on several factors such as genotype [15], bird's activity on the shackle line [16], stunning and bleeding efficiency [17]. The economic value of the haplotype presently identified for meat-type chicken selection will have to be studied more extensively by estimating the frequency of alternative alleles in commercial populations and their effect on color and other important economic traits. Any significant effect of the haplotype on body weight as well as breast or abdominal fatness percentage was observed in the F2 experimental cross which suggested that the effect of the mutation was specific to meat color. Fast genetic tests for the two SNPs by High Resolution Melting method have been developed (for an illustration of the results obtained for SNP2 see Figure S2) and make the association studies possible in various populations. Interestingly, the genotyping of a first commercial population showed that this polymorphism was not specific to the divergent HG and LG lines. The effect of the haplotype on the color of the meat was also significant in this commercial line.

Several mutations have been found in the coding region of *BCMO1* gene in humans. One, most likely very rare, leads to a drastic enzyme loss-of-function (90%) and hypercarotenemia and hypovitaminosis A [18]. Others, more frequently distributed also reduce enzyme activity but to a lower extent [19]. *BCMO1* knock-out in mice had no evident effects on growth but revealed, as expected, a dramatic decrease of vitamin A level in several tissues [20]. Liver steatosis developed without an increase in liver fatty acid synthase, independently of dietary vitamin A levels. Serum free fatty acid levels increased in *BCMO1* ^−/−^ mice, whereas no changes in serum glucose level were noticed. In response to dietary oil supplementation (30%), *BCMO1* ^−/−^ mice became heavier. In our chicken model, in addition to be of lower weight, LG chickens carrying the “deficient” *BCMO1* promoter gene are leaner and exhibit higher plasma glucose but normal free fatty acid levels as compared to HG chickens [21]. As body weight and metabolic parameters are under the control of many loci that are segregating between the two divergent HG and LG lines [21], further investigations using *BCMO1* mutant chickens with a common genetic background will be needed to precise the specific effects of that gene and to know whether chicken and mice respond differently to variations in *BCMO1* activity.

The color of chicken skin, easily visible at the level of the shank, is also dependent upon dietary carotenoid levels and carotenoid metabolism. Interestingly, a recent study [22] showed that the yellow skin phenotype is caused by mutation(s) in the tissue-specific regulatory elements of *BCMO2* gene (formerly called *BCDO2*) which encodes β-carotene 9′, 10′-monooxygenase. These mutations prevent expression of the gene in the skin but not in liver. While *BCMO1* and *BCMO2* have close functions (the first monooxygenase being responsible for the centric cleavage of the β-carotene and the second for its eccentric cleavage), they are located on different chromosomes (GGA11 and GGA24, respectively). Our findings open the way to future research on the specific or combined role of the two genes on color variation in chicken and in other species.

## Materials and Methods

### Ethics Statement

Chickens were bred at INRA, UE1295 Pôle d'Expérimentation Avicole de Tours, F-37380 Nouzilly in accordance with European Union Guidelines for animal care and under authorization 37-123 delivered to E. Le Bihan-Duval by the French Ministry of Agriculture. Animal procedure was approved by Departmental Direction of Veterinary Services of Indre-et-Loire.

### Animals and Color Measurements

The HG and LG chicken lines have been divergently selected for BW for more than 20 generations [1]–[3]. Characteristics of the F2 experimental cross (N = 698) used for QTL analysis have been described in detail previously [7]. Breast meat color of 9 week old birds was measured 24 h post-slaughter on the upper ventral side of the P. *major* muscle using a Miniscan Spectrocolorimeter. Color was measured by the CIELAB system, where L* represents lightness, a* redness and b* yellowness. Higher L*, a* and b* values correspond to paler, redder and more yellow meat, respectively.

### Genetic Markers

Genomic DNA was extracted from whole blood and QTL mapping on *GGA*11 was originally developed with three polymorphic microsatellite markers (MCW097, ADL210 and ADL308) [7]. A re-analysis of the meat color QTL on *GGA*11 was performed by adding three additional markers (SEQALL0528, SEQALL0529 and MCW230). These markers were already available [23] and were selected for their informativeness among microsatellite markers developed from the chicken genome assembly [21]. Fluorescently-labeled microsatellite markers were analyzed on an ABI 3700 DNA Analyzer (Applied Biosystems, Foster City, CA USA) and genotypes were called using GeneMapper software (Applied Biosystems, Foster City, CA USA). All F0, F1 and F2 (males and females) animals were genotyped with the six microsatellite markers.

### QTL Mapping

Prior to QTL detection, the data were corrected for sex and hatch effects as estimated by PEST software [24]. QTL detection, with both F2 and half sib analyses, were conducted by QTL Express, a web based software program [25]. Permutations were done with 10,000 iterations to obtain an empirical threshold at the chromosome level [26]. The 95% confidence interval for a QTL position was estimated by the two LOD (logarithm of the odds) drop-off method [27].

### Measurement of *BCMO1* mRNA Levels

The expression of *BCMO1* gene was first measured in the *Pectoralis major* muscle of six HG and LG individuals slaughtered at 1, 3, 5, 7, 9 and 11 weeks of age. Then to test the association between SNP mutations and *BCMO*1 expression, *BCMO1* mRNA levels were measured on 373 F2 birds from the HG x LG intercross, including all 134 offspring of the F1 sire family used for eQTL detection and additional 239 birds issued from the other F1 sire families. Methods used for preparation of RNA samples and quantitative real-time RT-PCR assay were described earlier [28]. The chicken *BCMO1* sequence (143 bp spanning exon 1 and 2) was amplified using 5′-GCCAAGCCATCAAACCAGTG-3′ and 5′-AACAAAGAAGAGCATCCAGAGCC-3′ as reverse and forward primers, respectively. The mRNA levels were estimated based on the cycle threshold (CT) deviation of an unknown sample versus a control cDNA (consisting of a pool of chicken muscle cDNA) according to the equation proposed by Pfaffl [29] and the data were expressed as dCT BCMO1 or dCT BCMO1-dCT 18S.

### DNA Sequencing

PCR primers were designed to amplify the 11 *BCMO1* exons and the putative promoter region from the chicken genome assembly (http://genome.ucsc.edu/index.html) using Lightcycler Probe Design 2 software (Roche) (See Table S1). PCR amplifications were carried out for each marker and each F1 sire in a 15 µl reaction containing 25 ng DNA, 0.4 µM primers, 0.25 units Taq polymerase (Go Taq, Promega), 1X buffer (Promega), 2 mM MgCl_2_ and 0.2 mM dNTP on a GeneAmp® PCR System 9700 thermocycler (Applied Biosystems). The first 5-min denaturation was followed by 38 cycles of: denaturation at 94°C for 30 sec, annealing for 30 sec and elongation at 72°C for 30 sec, with a final elongation of 10 min at 72°C. After Exo/SAP-IT (USB Europe GmbH, Staufen, Germany) purification, PCR products were sequenced on both strands using the same primers and a Big Dye Terminator V3.1 kit (Applied Biosystems) on an ABI 3100 DNA Analyzer (Applied Biosystems). All new polymorphisms have been deposited in dbSNP (http://www.ncbi.nlm.nih.gov/projects/SNP/).

### Genotyping

Candidate mutations were screened by high-resolution melting curve (HRM) analysis (see Table S2 for primers and probes). For SNP1 analysis, PCR amplifications were carried out in a 15 µl reaction containing 25 ng DNA, 0.5 µM primer 1, 0.1 µM primer 2, 0.25 units Taq polymerase (Go Taq, Promega), 1X buffer, 2 mM MgCl_2_ and 0.2 mM dNTP on a GeneAmp® PCR System 9700 thermocycler (Applied Biosystems). The first 5-min denaturation was followed by 45 cycles, each of denaturation at 95°C for 20 sec, annealing at 58°C for 20 sec and elongation at 72°C for 20 sec, with a final elongation for 10 min at 72°C. After addition of 0.4 µM of allele-specific probe and 1X LC Green (Idaho Technology) to 8 µL of PCR products, the samples were heated to 95°C for 10 min and then progressively cooled to 60°C by 5°C increments and then to 40°C by 10°C increments, 10 sec for each step during, with a ramp rate of 2.2°C/sec, on a LightCycler 480 (Roche). Then the mix was heated to 95°C (0.02°C/sec). The fluorescence data were subsequently analyzed by LightCycler 480 Gene scanning software. SNP2 analysis was performed similarly, except probe and LightCycler Green were added to the PCR reaction mix, and the amplification and HRM analyses were realized on the LightCycler 480: the first 10-min denaturation was followed by 50 cycles, each of denaturation at 95°C for 1 s, annealing at 58°C for 1 sec and elongation at 72°C for 10 sec; then the PCR products were heated to 95°C for 1 sec, and the dissociation curve was obtained by cooling to 40°C and heating to 95°C at a ramp rate of 0.02°C/sec.

### Muscle carotenoid measurements

Muscle carotenoid contents were compared between homozygous birds from the F2 population (N = 9 and 11 for AN_57_A and GN_57_G, respectively). Birds from the two genotypes were chosen within the same sire family and exhibited significant differences in *BCMO1* expression (1.7 *vs* -1.3 on the dCT scale for AN_57_A and GN_57_G, respectively) and yellow breast meat color (9.5 *vs* 12.7 for AN_57_A and GN_57_G, respectively). Carotenoid concentrations were determined in *Pectoralis major* muscle by using Ultra Performance Liquid Chromatography (UPLC) equipped with a 150×2.1 mm Acquity UPLC HSS T3, 1.8 µm column and a 2996 PDA detector (Waters, Saint-Quentin-en-Yvelines, France) according to an initial procedure used for plasma by Chauveau-Duriot et al. [30] adapted to muscle samples. Carotenoids were extracted by hexane (2×4 ml) from around 0.8 g of muscle sample after ethyl alcohol deproteinisation. Sample treatments were performed at room temperature under yellow light, with echinenone as the internal standard. Lutein, zeaxanthin and all-trans β-carotene were detected at 450 nm and identified by comparison of retention times and spectral analyses with those of pure standards (>95%) purchased from Carotenature (Lupsingen, Switzerland). Concentrations of carotenoids were calculated by using an external standard curve and then were adjusted by percent recovery of the added internal standard.

### Study of an unrelated commercial population

Birds originated from a slow-growing commercial line selected by a breeding company since 1994. A large pedigree population was constituted with 868 male and female birds issued from 10 sires and 100 dams. Birds were reared in three successive batches under similar conditions of free-range chickens with access to outdoor area after 6 weeks of age. At 12 weeks of age, birds were slaughtered after a 7 hours feed withdrawal. As for the experimental population, color was measured on the upper ventral side of the P. *major* muscle by using a Miniscan spectrocolorimeter. It was measured in the CIELAB trichromatic system by Lightness (L*), redness (a*) and yellowness (b*). All the sires and dams were genotyped as described previously. In a second step, genotyping was performed on the offspring of the heterozygous sires (N = 470). *BCMO1* gene expression was measured in a sub-population of 11 AN_57_A and 12 GN_57_G homozygous male birds chosen within one batch of hatching.

### Plasmid Constructions

Chicken *BCMO*1 promoter with the GN_57_G haplotype was amplified by PCR from bases –734 to + 97 relative to the transcription start site from exon 1, using the following primers: BCMOF: 5′-AGCCTGTGATTTCCTCTGC-3′ and BCMO1R: 5′-TCGCTCTCCTGTGTCCTCTA-3′. First, this fragment was subcloned into PCR3.1 plasmid (Invitrogen, Carlsbad, CA) by TA cloning procedure. The promoter insert of PCR3.1 plasmid was then cloned into pGL2 basic plasmid (Promega, Madison, WI), upstream of the luciferase reporter gene, to create pGL2-BCMO1-Luc plasmid. Site-directed mutagenesis were performed with the QuikChange II Site-Directed Mutagenesis Kit (Stratagene) using the following mutagenic primers (only forward primers are shown), 5′-AATCCCCCTTCTTCTTAATTATTCCT**A**CCATACTTTCGCAGAGG-3′ and 5′-GTGGTGGAATCAGATCACACGT**A**GGAGAATGTAGAATAGTGCC-3′ (the mutated bases are in bold) to create the AN_57_A, AN_57_G and GN_57_A haplotypes.

#### Cell culture and Transactivation Assay

The chicken hepatocellular carcinoma cell line (LMH) was obtained from ATCC (cat # CRL-2117). LMH cells were grown in Waymouth's medium (Invitrogen, Carlsbad, CA) supplemented with 10% foetal calf serum (FCS, Invitrogen, Carlsbad, CA), 100 IU/ml penicillin (Invitrogen, Carlsbad, CA), and 100 mg/ml streptomycin (Invitrogen, Carlsbad, CA). The human hepatocellular carcinoma cells (HuH-7) were provided by the unit 6061-INSERM-France. HuH-7 cells were grown in William's medium (Invitrogen, Carlsbad, CA), 10% FCS, 1.6 ml BSA 30%, 500 nM Hydroxy-Cholesterol (GE Healthcare, Piscataway, NJ), 1 µg/mL Insuline (SIGMA, St. Louis, MO). All cultures were incubated at 37°C in a humidified atmosphere containing 5% CO_2_. For transactivation assay, transient transfections of LMH cells were performed in triplicate in 6 well plates using the FuGENE lipofection protocol (Roche, Neuilly sur Seine, France). Each well was transfected with 200 ng of pGL2-BCMO1-Luc reporter plasmid and 20 ng of pCMV-bgal reference plasmid containing a bacterial β-galactosidase gene. After 24 h following transfection, the cells were washed once with phosphate buffered saline, and incubated with fresh medium. After 48 h following transfection, the cells were lysed (Lysis buffer - Promega, Madison, WI). Cell lysates were stored at -80°C before analyses. After centrifugation 5 min at 12000 *g,* supernatants (10 µl) were incubated 5 s in the presence of 40 µl of luciferase assay buffer (Promega, Madison, WI) including luciferin (470 µM). Luciferase activity was determined using a luminometer (Veritas Turner Biosystems, Sunnyvale, XA). The β-galactosidase activity was measured by hydrolysation of Ortho Nitro Phenyl Galactopyranoside (ONPG, SIGMA, St. Louis, MO) and used to normalize the luciferase activity.

## Supporting Information

Figure S1 Polymorphisms within the BCMO1 promoter sequence (-817 to +41 bp). All the SNPs found in at least one HG x LG F1 sire are italicized (light red font), while the two linked candidate SNP are in red bold font and underlined (SNP1: CTG/ACC, SNP2: GTG/AGG). The ATG codon is shown as a shaded box.
(0.33 MB TIF)

Figure S2 Melting curves obtained for SNP2 (GTG/AGG). The homozygous AN57A are characterized by a fluorescence peak at 63°C (blue line), the homozygous GN57G by a fluorescence peak at 68°C (red line) and the heterozygous by two fluorescence peaks at 63°C and 68°C (green line).
(0.32 MB TIF)

Table S1 Primers and PCR conditions used to amplify the BCMO1 promoter region and 11 exons.
(0.04 MB DOC)

Table S2 Primers and probes used to perform HRM curve analyses.
(0.03 MB DOC)

## References

[1] Ricard FH (1975). Essai de sélection sur la forme de la courbe de croissance chez le poulet: Dispositif expérimental et premiers résultats.. Ann Génét Sél Anim.

[2] Mignon-Grasteau S (1999). Genetic parameters of growth curve parameters in male and female chickens.. Brit Poult Sci.

[3] Mignon-Grasteau S, Beaumont C, Ricard FH (2001). Genetic analysis of a selection experiment on the growth curve of chickens.. Poult Sci.

[4] Beccavin C, Chevalier B, Cogburn LA, Simon J, Duclos MJ (2001). Insulin-like growth factor and body growth in chickens divergently selected for high or low growth rate.. J Endocrinol.

[5] Duclos MJ, Chevalier B, Rémignon H, Ricard FH, Goddard C (1996). Divergent selection for high or low growth rate modifies the response of muscle cells to serum or Insulin-like growth factor-I in vitro.. Growth Reg.

[6] Rémignon H, Gardahaut MF, Marché G, Ricard FH (1995). Selection for rapid growth increases the number and the size of muscle fibres without changing their typing in chickens.. J Muscle Res Cell Motil.

[7] Nadaf J, Gilbert H, Pitel F, Berri CM, Feve K (2007). Identification of QTL controlling meat quality traits in an F2 cross between two chicken lines selected for either low or high growth rate.. BMC Genomics.

[8] Biesalski HK, Chichili GR, Frank J, Von Lintig J, Nohr D (2007). Conversion of β-carotene to retinal pigment.. Vitamin Horm.

[9] Georges M (2007). Mapping, fine mapping, and molecular dissection of quantitative trait Loci in domestic animals.. Ann Rev Genomics Hum Genet.

[10] Ron M, Weller JI (2007). From QTL to QTN identification in livestock – winning by points than knock-out: a review.. Animal Genetics.

[11] Fletcher DL (1999). Poultry Meat colour..

[12] Lietz G, Lange J, Rimbach (2010). Molecular and dietary regulation of β,β-carotene 15,15′ monooxygenase 1 (BCMO1).. Arch Biochem Biophys.

[13] Moren M, Naess T, Hamre K (2002). Conversion of β-carotene, canthaxanthin and astaxanthin to vitamin A in Atlantic halibut (*Hippoglossus hippoglossus* L.) juveniles.. Fish Physiol Biochem.

[14] Sangeetha RK, Baskaran V (2010). Retinol-deficient rats can convert a pharmacological dose of astaxanthin to retinol: antioxidant potential of astaxanthin, lutein and β-carotene.. Can J Physiol Pharmacol.

[15] Berri C, Wacrenier N, Millet N, Le Bihan-Duval E (2001). Effect of selection for improved body composition on muscle and meat characteristics of broilers from experimental and commercial lines.. Poult Sci.

[16] Berri C, Debut M, Santé-Lhoutellier V, Arnould C, Boutten B (2005). Variations in chicken breast meat quality: implications of struggle and muscle glycogen content at death.. Brit Poult Sci.

[17] Alvarado CZ, Richards MP, O'Keefe SF, Wang H (2007). The Effect of blood removal on oxidation and shelf life of broiler breast meat.. Poult Sci.

[18] Lindqvist A, Sharvill J, Sharvill DE, Andersson S (2007). Loss-of-Function Mutation in Carotenoid 15,15′-Monooxygenase Identified in a Patient with Hypercarotenemia and Hypovitaminosis A.. J Nutr.

[19] Leung WC, Hessel S, Méplan C, Flint J, Oberhauser V (2009). Two common single nucleotide polymorphisms in the gene encoding beta-carotene 15,15′-monoxygenase alter beta-carotene metabolism in female volunteers.. FASEB J.

[20] Hessel S, Eichinger A, Isken A, Amengual J, Hunzelmann S (2007). CMO1 deficiency abolishes vitamin A production from beta-carotene and alters lipid metabolism in mice.. J Biol Chem.

[21] Nadaf J, Pitel F, Gilbert H, Duclos MJ, Vignoles F (2009). QTL for several metabolic traits map to loci controlling growth and body composition in an F2 intercross between high- and low-growth chicken lines.. Physiol Genomics.

[22] Eriksson J, Larson G, Gunnarsson U, Bed'hom B, Tixier-Boichard M (2008). Identification of the Yellow Skin Gene Reveals a Hybrid Origin of the Domestic Chicken.. PLoS Genet.

[23] Groenen MA, Cheng HH, Bumstead N, Benkel BF, Briles WE (2000). A consensus linkage map of the chicken genome.. Genome Res.

[24] Groeneveld E (1990). PEST User's Manual.. Germany: Institute of Animal Husbandry and Animal Behaviour, Federal Agricultural Research Center (FAL), W 3057 Neustadt 1, Hoeltystre10.

[25] Seaton G, Haley CS, Knott SA, Kearsey M, Visscher PM (2002). QTL Express: mapping quantitative trait loci in simple and complex pedigrees.. Bioinformatics.

[26] Churchill GA, Doerge RW (1994). Empirical threshold values for quantitative trait mapping.. Genetics.

[27] Mangin B, Goffinet B (1997). Comparison of several confidence intervals for QTL location.. Heredity.

[28] Sibut V, Le Bihan-Duval E, Tesseraud S, Godet E, Bordeau T (2008). Adenosine monophosphate-activated protein kinase involved in variations of muscle glycogen and breast meat quality between lean and fat chickens.. J Anim Sci.

[29] Pfaffl MW (2001). A new mathematical model for relative quantification in real-time RT-PCR.. Nucleic Acids Res.

[30] Chauveau-Duriot B, Doreau O, Nozière P, Graulet B (2010). Simultaneous quantification of carotenoids, retinol, and tocopherols in forages, bovine plasma, and milk: validation of a novel UPLC method.. Anal Bioanal Chem.

