# Correction: Detection of a Cis eQTL Controlling *BMCO1* Gene Expression Leads to the Identification of a QTG for Chicken Breast Meat Color

**DOI:** 10.1371/annotation/17f0c1e7-7cca-4886-9f0b-3891ab2dc128

**Published:** 2011-09-23

**Authors:** Elisabeth Le Bihan-Duval, Javad Nadaf, Cécile Berri, Frédérique Pitel, Benoît Graulet, Estelle Godet, Sophie Y. Leroux, Olivier Demeure, Sandrine Lagarrigue, Cécile Duby, Larry A. Cogburn, Catherine M. Beaumont, Michel J. Duclos

The name of the gene (BCMO1) was spelled incorrectly in the title.

The title should be: Detection of a Cis eQTL Controlling BCMO1 Gene

Expression Leads to the Identification of a QTG for Chicken Breast Meat Color

The corrected citation is: Le Bihan-Duval E, Nadaf J, Berri C, Pitel F, Graulet B, et al. (2011) Detection of a Cis eQTL Controlling BCMO1 Gene Expression Leads to the Identification of a QTG for Chicken Breast Meat Color. PLoS ONE 6(7): e14825. doi:10.1371/journal.pone.0014825 

