# Supplementary figures and images for: Detection of a Cis eQTL Controlling BMCO1 Gene Expression Leads to the Identification of a QTG for Chicken Breast Meat Color
Source: PLoS One. 2011 Jul 5;6(7):e14825. doi: 10.1371/journal.pone.0014825 (PMC3130028; doi:10.1371/journal.pone.0014825)

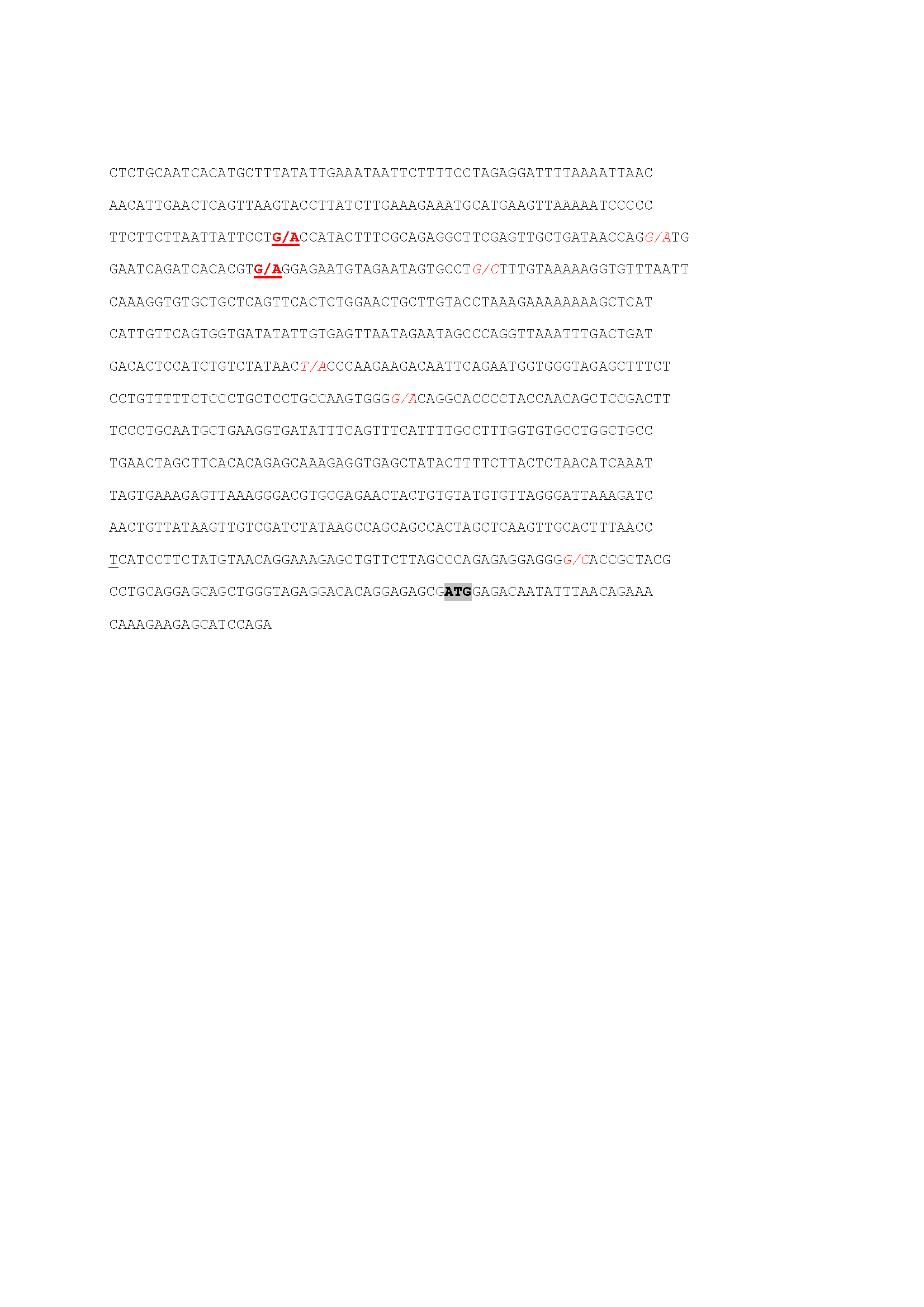

Supplement: Figure S1 — Polymorphisms within the BCMO1 promoter sequence (-817 to +41 bp). All the SNPs found in at least one HG x LG F1 sire are italicized (light red font), while the two linked candidate SNP are in red bold font and underlined (SNP1: CTG/ACC, SNP2: GTG/AGG). The ATG codon is shown as a shaded box. (0.33 MB TIF) [file pone.0014825.s001.tif]

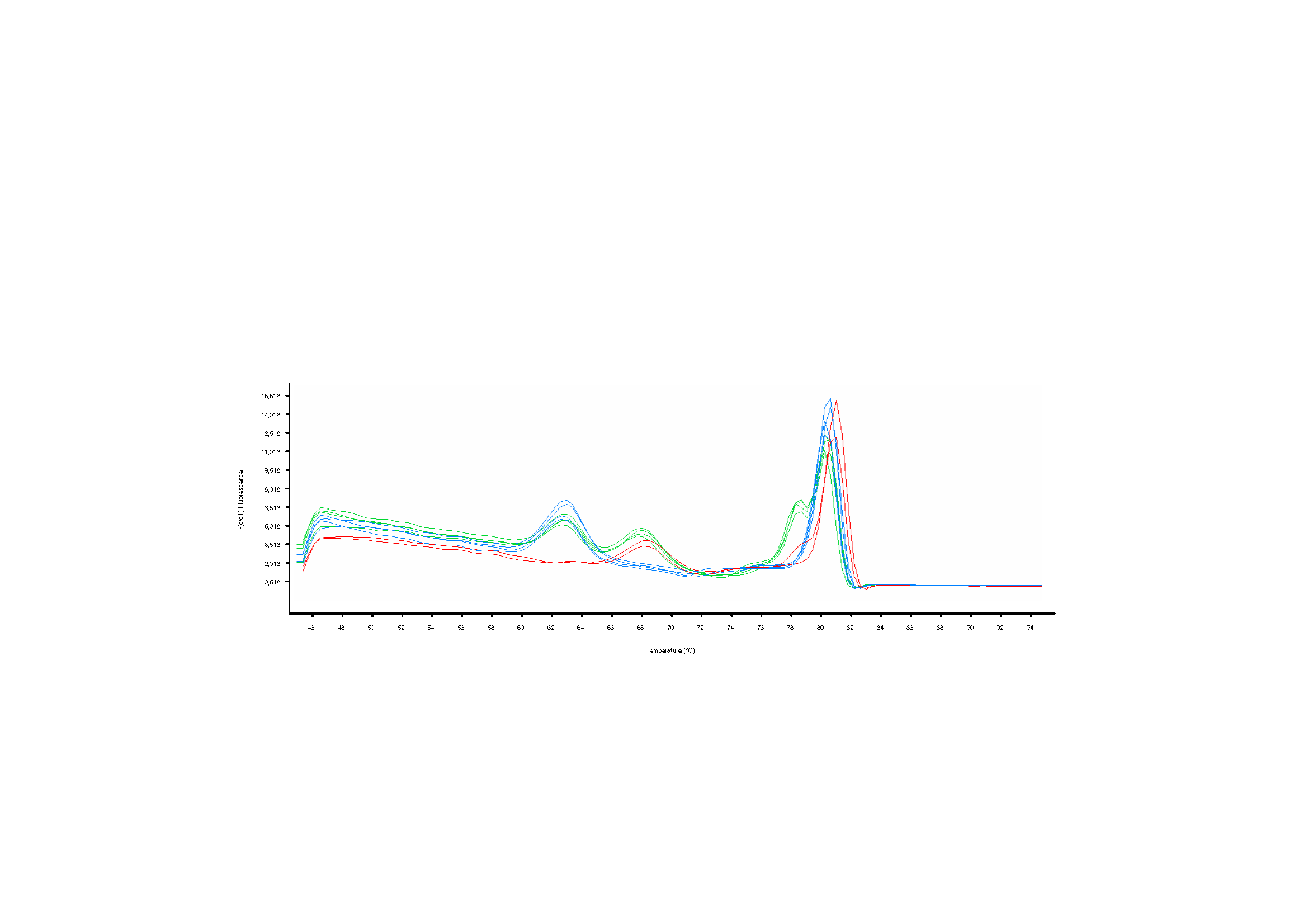

Supplement: Figure S2 — Melting curves obtained for SNP2 (GTG/AGG). The homozygous AN57A are characterized by a fluorescence peak at 63°C (blue line), the homozygous GN57G by a fluorescence peak at 68°C (red line) and the heterozygous by two fluorescence peaks at 63°C and 68°C (green line). (0.32 MB TIF) [file pone.0014825.s002.tif]
