# Supplementary material for: Detection of a Cis eQTL Controlling BMCO1 Gene Expression Leads to the Identification of a QTG for Chicken Breast Meat Color
Source: PLoS One. 2011 Jul 5;6(7):e14825. doi: 10.1371/journal.pone.0014825 (PMC3130028; doi:10.1371/journal.pone.0014825)
Supplement: Table S1 — Primers and PCR conditions used to amplify the BCMO1 promoter region and 11 exons. (0.04 MB DOC) [file pone.0014825.s003.doc]

| **Amplified region** | **Primer 1** | **Primer 2** | **Size (bp)** | **PCR Tm** |
| --- | --- | --- | --- | --- |
| promoter | CTCTGCAATCACATGCTTTAT | TCTGGATGCTCTTCTTTGTTT | 858 | 52°C |
| exon 1 | AAGATCAACTGTTATAAGTTGTCG | CTGCAAGTATTAGAAGAGATTGTCA | 261 | 55°C |
| exon 2 | TATGAAGCCTATGCCCTTAGT | TGAATTGGGAACATAAGACACC | 286 | 55°C |
| exon 3 | GAAACGTAGTGTGAAATGTGAT | ATATTTGTATTCACGTGCCAAGA | 270 | 55°C |
| exon 4 | CTGCAGGTACAGCTTCT | GCCAGGAAATGGGAGGAAATA | 302 | 55°C |
| exons 5 and 6 | ATGGGCATTTGCAGAGT | GAGCAGCAACAGTAACAAC | 842 | 55°C |
| exon 7 | ATTTCCTATGTAACTGTTTGCTG | CAACTGCTCATCCACCC | 403 | 55°C |
| exon 8 | ATTAGAAGCACATTACATTTCCAG | ACATCTAAGTGTAGGTGTACAAG | 204 | 52°C |
| exon 9 | TACCACTTTTGAACAAACTGCC | TCTGGGTTGGTGACTGCAGA | 423 | 55°C |
| exon 10 | ACAATGTAATGAAGTGGTATCTATG | TGCAAGCTCCTTTTGCTC | 538 | 55°C |
| exon 11 | AGATTCCTAGCAAACAAGACT | TTTGTGTCGTTATATGGTTGTTC | 366 | 52°C |
