# Supplementary material for: Detection of a Cis eQTL Controlling BMCO1 Gene Expression Leads to the Identification of a QTG for Chicken Breast Meat Color
Source: PLoS One. 2011 Jul 5;6(7):e14825. doi: 10.1371/journal.pone.0014825 (PMC3130028; doi:10.1371/journal.pone.0014825)
Supplement: Table S2 — Primers and probes used to perform HRM curve analyses. (0.03 MB DOC) [file pone.0014825.s004.doc]

| **Marker** | **Primer 1** | **Primer 2** | **Probe** |
| --- | --- | --- | --- |
| SNP1 | GAACTCAGTTAAGTACCTTATCT | AGCAGCACACCTTTGAATTAAACA | GCGAAAGTATGGCAGGAATAATTAA |
| SNP2 | AGGCTTCGAGTTGCTGATAACC | AGCAGCACACCTTTGAATTAAACA | CTATTCTACATTCTCCCACGTGTGATCTGATT |
